# Inflammation-induced fetal growth restriction in rats is associated with increased placental HIF-1α accumulation

**DOI:** 10.1371/journal.pone.0175805

**Published:** 2017-04-19

**Authors:** Kevin P. Robb, Tiziana Cotechini, Camille Allaire, Arissa Sperou, Charles H. Graham

**Affiliations:** Department of Biomedical and Molecular Sciences, Queen’s University, Kingston, Ontario, Canada; Universidade de Sao Paulo Instituto de Ciencias Biomedicas, BRAZIL

## Abstract

**Introduction:**

Hypoxia-inducible factor 1-alpha (HIF-1α) is the oxygen-sensitive subunit of the transcription factor HIF-1, and its expression is increased in placentas from pregnancies complicated by pre-eclampsia (PE). Fetal growth restriction (FGR) and PE often share a common pathophysiology; however, it is unknown whether increased placental HIF-1α occurs in FGR. We previously demonstrated that aberrant maternal inflammation in rats resulted in altered utero-placental perfusion and FGR, both of which were prevented by administration of the nitric oxide mimetic glyceryl trinitrate (GTN). Our aim here was to determine whether abnormal maternal inflammation causing FGR is linked to placental HIF-1α accumulation and whether GTN administration could prevent increases in placental HIF-1α.

**Methods:**

Levels of inflammatory factors in maternal plasma were measured using a multiplex assay after an injection of low-dose lipopolysaccharide (LPS) to rats on gestational day (GD) 13.5. Following three additional daily LPS injections from GD14.5–16.5, GD17.5 placentas were harvested for HIF-1α immunolocalisation; serial sections were also stained for the hypoxia marker pimonidazole. A subset of rats received LPS injections along with GTN delivered continuously (25 μg/h via a transdermal patch) on GD12.5-GD17.5.

**Results:**

Within two hours of LPS administration, levels of maternal pro-inflammatory cytokines were increased compared with saline-treated controls. GD17.5 placentas of growth-restricted fetuses exhibited increased HIF-1α accumulation; however, this did not correlate with pimonidazole staining for which no differences were observed between groups. Furthermore, the LPS-mediated increases in maternal inflammatory cytokine levels and placental HIF-1α accumulation did not occur in rats treated with GTN.

**Discussion:**

Our results demonstrate that inflammation-induced FGR is associated with increased placental HIF-1α accumulation; however, expression of this transcription factor may not correlate with regions of hypoxia in late-gestation placentas. The GTN-mediated attenuation of placental HIF-1α accumulation in LPS-treated rats provides insight into the mechanism by which GTN improves inflammation-induced complications of pregnancy.

## Introduction

Hypoxia-inducible factor 1 (HIF-1) is a transcription factor consisting of an alpha (α) subunit, normally stabilized under hypoxic conditions, and a constitutively-expressed beta (β) subunit. Pathological expression of HIF-1α is associated with pre-eclampsia (PE), a complication of pregnancy linked to deficient remodelling of the maternal spiral arteries and altered utero-placental perfusion [1]. It has been reported that HIF-1α is over-expressed in placentas from pre-eclamptic pregnancies and that it fails to be down-regulated after the first trimester [2, 3] when placental oxygenation normally increases [4]. Fetal growth restriction (FGR; the failure of a fetus to achieve its genetically pre-determined growth) often occurs with PE and is also associated with altered utero-placental perfusion [5]; however, whether HIF-1α over-expression occurs in the placentas of growth restricted fetuses has not been investigated.

Inflammation is thought to play a central role in the pathophysiology of spontaneous pregnancy loss (SPL), PE, and FGR with several studies reporting increased levels of inflammatory cytokines and chemokines systemically as well as locally within the placenta [6–14]. Using a model of SPL in which rats are injected with a single high-dose of the endotoxin lipopolysaccharide (LPS), we demonstrated that aberrant maternal inflammation was associated with altered utero-placental perfusion and placental hypoxia [15]. In our more recent study, pregnant rats were administered chronic low-doses of LPS to induce a less severe inflammatory response; this instead resulted in FGR and a maternal syndrome with characteristics of PE that included renal histopathological abnormalities, proteinuria, impaired spiral artery remodelling and altered utero-placental hemodynamics [16]. Using this rat model, we have also reported that inflammation-induced FGR is associated with altered placental morphometrics including reduced placental weight and thickness, as well as decreased placental area [17]. Taken together, these results are suggestive of a potential link between aberrant inflammation, inadequate utero-placental perfusion, and decreased oxygen tension within the placenta, which in turn may lead to the HIF-1α accumulation often observed in PE. However, other factors including reactive oxygen species and inflammation-induced NF-κB have been shown to modulate HIF-1α accumulation [18, 19], and it is not known which of these contribute under pathological conditions in the placenta.

Increased HIF-1α expression has been linked to dysregulation of genes important to normal placental function [20]; therefore, determining whether HIF-1α levels are increased in placentas associated with FGR and/or the mechanism for increased placental HIF-1α may be important for the development of novel therapeutic strategies. FGR affects 5–8% of pregnancies and is the second leading cause of perinatal mortality [21, 22]; however, at present there is no effective therapy for this condition. To address this, our laboratory has investigated the use of the nitric oxide (NO) mimetic glyceryl trinitrate (GTN) as a potential therapeutic agent in the treatment of FGR and PE. Through the culture of human term chorionic villi explants, we showed that GTN treatment inhibited syncytiotrophoblast apoptosis following cycles of hypoxia/reoxygenation [23]. In a follow up study, we reported that GTN impaired hypoxia-induced HIF-1α accumulation in chorionic villi explants, as well as the release of downstream soluble fms-like tyrosine kinase 1 (sFlt-1) and soluble endoglin (sEng) [24], key mediators of the endothelial dysfunction characteristic of FGR and PE [23, 25–27]. In our inflammation-induced rat model of FGR/PE, we also demonstrated that transdermal administration of GTN resulted in normalisation of utero-placental perfusion as well as prevention of maternal coagulopathies, placental nitrosative stress, fetal loss, proteinuria and increases in maternal blood pressure [16, 28]. Taken together, these results advocate for the continued study of NO mimetics as potential drugs for the treatment of pathological pregnancies linked to aberrant maternal inflammation.

In the present study we used our inflammation-induced rat model of FGR/PE [16] to determine whether FGR is associated with augmented placental HIF-1α accumulation and whether HIF-1α accumulation in the placenta is linked to hypoxia. Since GTN prevented alterations in utero-placental perfusion and FGR in the same rat model used here [16], we also determined whether GTN could attenuate LPS-mediated increases in circulating inflammatory mediators and placental HIF-1α.

## Methods

### Experimental protocol

Treatment of rats was conducted in accordance with the Queen’s University Animal Care Committee and following the approval by this institutional committee (protocol number 2014–008). Rats were euthanized using an overdose of sodium pentobarbital (intraperitoneal injection; 0.44 mg/kg). We used our previously described methods for LPS and GTN administration in which we reported that LPS treatment during pregnancy led to FGR (~40% FGR pups/litter) and features of PE [16]. That study showed that LPS significantly reduced fetal weight compared to saline controls (0.8421 ± 0.006 g vs. 0.9244 ± 0.007 g) and that GTN treatment led to a significant increase in fetal weight (0.8794 ± 0.007 g) compared to LPS treatment alone. Briefly, virgin female Wistar rats (3–6 months old; Charles River Laboratories, Montréal, Canada) were mated overnight; presence of spermatozoa in the vaginal lavage marked gestational day (GD) 0.5. Pregnant rats received an initial intraperitoneal injection of LPS (*Escherichia coli* serotype 0111:B4; Sigma-Aldrich, Oakville, Canada; 10 μg/kg) or saline (1 ml/kg) on GD 13.5. Subsequent intraperitoneal injections of LPS (40 μg/kg) were performed once daily between GD 14.5 and 16.5. Dams were sacrificed on either GD 13.5, two hours after the initial LPS/saline injection (for multiplex analysis), or on GD 17.5, 24 hours after the final LPS/saline injection (for tissue harvest and immunohistochemical analysis). To evaluate the role of GTN on LPS-induced cytokine release and placental HIF-1α accumulation, an additional cohort of pregnant rats was administered a transdermal GTN patch (Graceway Pharmaceuticals, Bristol, USA; delivering 25 μg of GTN per hour; changed daily) between GD 12.5–17.5.

In summary, rats were distributed in three treatment groups: LPS (n = 7 dams per timepoint), Saline (n = 3 dams per timepoint), or LPS+GTN (n = 3 dams per timepoint). More dams were included in the LPS treatment group because these rats had fewer viable implantation sites to analyse as previously reported [28], and of these, only a fraction of the viable pups was growth restricted [16]. Fewer dams were required in the LPS+GTN group as there were significantly fewer resorption sites in this group compared to either the saline or LPS-treated groups [28]. Some of the animals used in this study were from the same cohort of animals used in our previous publication [16].

### Maternal plasma sample collection

Maternal blood was obtained by cardiac puncture at the time of euthanasia on GD 13.5, two hours after the initial LPS or saline injection, or on GD 17.5. Blood was collected into tubes pre-filled with ethylenediaminetetraacetic acid (EDTA) and centrifuged (2,000 x g for 20 min). Plasma was removed and stored at -80°C until multiplex cytokine analysis or ELISA.

### Multiplex analysis of plasma cytokines and measurement of circulating sFlt-1 levels

Rat plasma samples from animals treated with LPS (n = 7), saline (n = 3), or LPS+GTN (n = 3) were analysed using a Bio-Plex Pro Rat Cytokine 24-plex Assay (Bio-Rad, Mississauga, Canada; cat. #171-K1001M) according to the manufacturer’s instructions. The levels of all 24 factors were evaluated in this study, but only those samples with cytokine concentrations above the detectable range were included for analysis. Therefore, we were unable to perform statistical analysis for the following factors: IL-1 Ra, IL-1 β, IL-4, IL-7, IL-17A, IL-12p70 and GROα.

sFlt-1 detection was performed on plasma samples obtained from LPS (N = 10 dams), saline (N = 8), and LPS+GTN (N = 8) treated dams on GD 17.5 using the Rat VEGFR1 ELISA (RayBiotech, Norcross, USA, cat. ELR-VEGFR1-1) according to the manufacturer’s instructions. Grubb’s outlier test was used to identify outliers in each group; one data point was subsequently removed from the LPS+GTN group.

### Assessment of placental hypoxia

To assess placental hypoxia, animals were injected intraperitoneally with pimonidazole hydrochloride (Hypoxyprobe-1^®^, Burlington, USA; 60 mg/kg), one hour prior to euthanasia. Pimonidazole is a commonly used marker for hypoxia that binds to thiol groups of proteins under hypoxic conditions (pO_2_ < 10mmHg) [29], forming adducts that can be detected by immunohistochemistry (described below).

### Immunolocalisation of HIF-1α and pimonidazole adducts

Immunohistochemistry was used to examine HIF-1α expression and the presence of pimonidazole adducts in rat placentas collected from GD 17.5 dams. Following euthanasia, implantation sites were harvested and bisected through the mesometrial triangle and placenta so that the centre of each utero-placental unit could be evaluated. Tissues were fixed in 4% paraformaldehyde (PFA) for a minimum of 24 hours before being transferred to 70% ethanol. Serial sections (5–6 μm) from paraffin-embedded tissues were then prepared for immunohistochemical staining. The percentage of areas stained positive for HIF-1α and pimonidazole were compared between FGR placentas from LPS-treated dams, and non-FGR placentas from the LPS, saline, and LPS+GTN treatment groups. Placentas from each dam were randomly selected for analysis; variability in the number of placentas was due to differences in the percentage of FGR within each dam and the extent of fetal death, as described previously [16, 28].

#### HIF-1α

Placental HIF-1α accumulation was compared between placentas of LPS/FGR (n = 1–5 placentas from each of four dams; 10 placentas total), non-growth restricted fetuses from LPS-treated dams (LPS/non-FGR) (n = 1 placenta from each of five dams), saline (n = 1–3 placentas from each of four dams; nine placentas total), and LPS+GTN (n = 2 placentas from each of three dams except where specified). Briefly, tissues were rehydrated using a graded ethanol series and subjected to heat-mediated antigen retrieval in citrate solution (DakoCytomation, Glostrup, Denmark; 20 min). Following blocking of endogenous peroxidase and incubation in normal horse serum (NHS), slides were incubated overnight (4°C) with anti-HIF-1α primary antibody (Novus Biological, Oakville, Canada, cat. NB100-105; 1:500 in 2% NHS). A biotinylated horse anti-mouse secondary antibody (Vector Laboratories, Burlington, Canada; 1:200 in 1% NHS) was then applied (30 min) followed by antigen detection using a Vectastain ABC Elite kit (Vector Laboratories; 45 min) and incubation with diaminobenzidine (DAB) solution (Dako North America, Burlington, Canada) for one minute. After detection, tissues were counterstained with hematoxylin (Fisher Scientific, Ottawa, Canada), dehydrated, and mounted. As a negative control, normal rabbit IgG (Dako North America) was used in place of the primary antibody at the same concentration.

#### Pimonidazole

To examine placental hypoxia, serial sections of placentas from dams injected with pimonidazole were analyzed. Staining for pimonidazole was performed according to the instructions of the manufacturer of the Hypoxyprobe^®^ kit and as reported previously [15] on placentas from LPS/FGR (n = 2 placentas from each of two dams), LPS/non-FGR (n = 1–2 placentas from each of two dams; three placentas total), Saline (n = 1–2 placentas from each of four dams; six placentas total), and LPS+GTN (n = 1–2 placentas from each of three dams; four placentas total). Antigen detection was performed using a ten-minute incubation with the 3-amino-9-ethylcarbazole (AEC) substrate system (Abcam, Cambridge, USA) followed by counterstaining with hematoxylin, dehydration, and mounting.

### Image analysis

Slides were scanned at 20× using an Aperio ImageScope system and software (Aperio, Vista, USA). To analyse HIF-1α staining, an algorithm that detects areas stained with DAB was used to quantify the number of pixels comprising moderate and strongly stained regions; this was divided by the total pixelated area of the implantation site to obtain the percentage of positively stained area. A positive pixel count was also used to quantify areas stained for pimonidazole with AEC; the percentage of positively stained area was calculated using the moderate and strongly stained areas.

### Statistics

Statistical analysis was performed using GraphPad Prism 6.0 Software (GraphPad, La Jolla, USA). Data from the cytokine multiplex and HIF-1α/pimonidazole immunohistochemistry were analysed using Kruskal Wallis followed by Dunn’s multiple comparison *post hoc* test. Multiplex data are presented as median (minimum—maximum). Data were considered significant when p<0.05, and trending toward significance when p<0.1. Spearman’s correlation was used to examine correlations between variables; the Spearman’s rank correlation coefficient, r_s_, and p values are reported for these analyses. Grubb’s test was used to identify statistical outliers.

## Results

### LPS increases the levels of maternal pro-inflammatory cytokines

Two hours after an initial injection of low-dose LPS (10 μg/kg) on GD 13.5, plasma levels of the inflammatory mediators tumour necrosis factor (TNF), interleukin (IL)-6, IL-10, monocyte chemotactic protein 1 (MCP-1) and granulocyte macrophage-colony stimulating factor (GM-CSF) were significantly increased compared with levels measured in plasma from saline-treated dams. In addition, levels of IL-5 and macrophage colony-stimulating factor (M-CSF) trended (p<0.1) towards being increased. Levels of LPS-induced inflammatory cytokines measured in plasma from dams pre-treated with GTN were not significantly different from levels measured in plasma from saline-treated controls or LPS-treated dams (Table 1).

**Table 1:** Inflammatory mediators measured in LPS, Saline and LPS+GTN treated pregnant rats.

	LPS (pg/mL)	Saline (pg/mL)	LPS+GTN (pg/mL)
**TNF**	2507.26* (55.22–12168.52)	51.09 (35.69–70.01)	407.18 (55.93–641.83)
**IL-5**	560.4ˆ (112.9–1650.74)	125.1 (101.2–226.5)	477.05 (127.42–553.39)
**IL-6**	6464.68* (786.4–20893.93)	21.42 (0–338.4)	924.11 (0.00–1809.41)
**IL-10**	3137.19* (565.5–6153.03)	270.2 (173.8–761.9)	1403.44 (358.89–3033.23)
**IL-13**	116.5 (13.29–208.2)	25.56 (12.76–61.82)	132.51 (19.84–134.97)
**MCP-1**	32048.63* (27859.25–44000)	525.3 (438.8–803.5)	22483.37 (891.21–22770.01)
**M-CSF**	1295.28^#^ (657.6–1679.68)	783.9 (584.9–851.2)	866.19 (771.67–980.88)
**GM-CSF**	128.6* (17.82–232.1)	16.62 (13.94–58.28)	110.38 (23.89–124.19)
**G-CSF**	22.58 (4.670–38.91)	4.28 (3.190–19.52)	24.19 (2.68–25.46)
**MIP-3α**	130.9 (74.93–721.4)	53.58 (19.91–103.5)	121.95 (47.52–325.17)
**VEGF**	101.7 (87.83–178.1)	90.30 (71.13–92.38)	100.37 (87.18–104.80)

Plasma from all rats was obtained 2h after LPS or saline administration; statistical comparisons between treatment groups were made using Kruskal Wallis followed by Dunn’s multiple comparison *post hoc* test. Data are presented as median (minimum—maximum). LPS: n = 7; Saline: n = 3; LPS+GTN: n = 3.

* p<0.05 versus Saline

ˆ p = 0.0.0767 versus Saline

^#^ p = 0.0803 versus Saline

LPS+GTN versus LPS and Saline: n.s.

### Placental HIF-1α accumulation is associated with FGR

**Fig 1:**
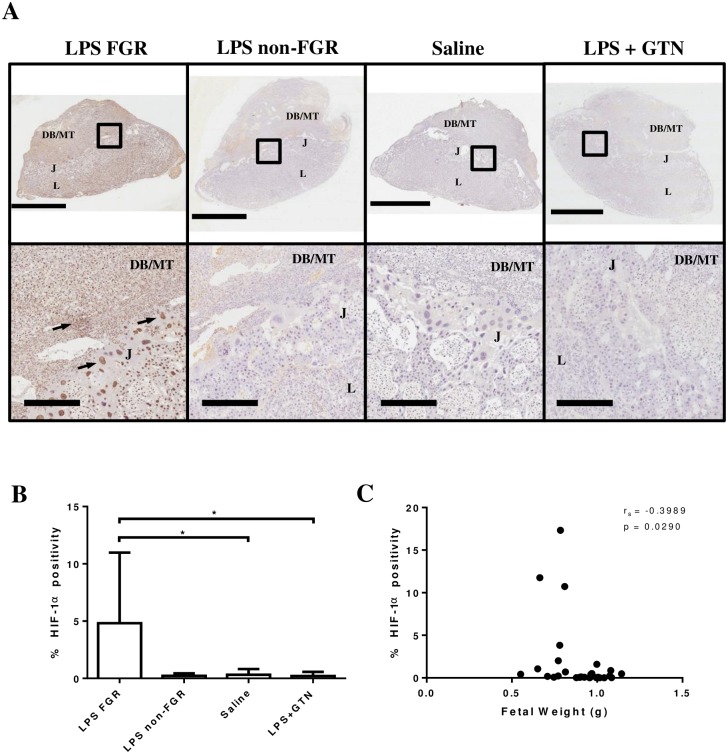
HIF-1α accumulation is increased in placentas of growth-restricted fetuses. Placentas from growth-restricted fetuses of LPS-treated dams (LPS FGR; n = 10 placentas) exhibited increased expression of HIF-1α (brown stain, arrows) (A) and significantly increased HIF-1α accumulation when compared with placentas of non-growth restricted fetuses from the saline (n = 9) and LPS+GTN (n = 5) treatment groups; LPS non-FGR (n = 5) (B). HIF-1α levels negatively correlated with fetal weight across all treatment groups (n = 30; r_s_ = -0.3989; p = 0.0290) (C). DB/MT, decidua basalis/mesometrial triangle; J, junctional zone; L, labyrinth. *p<0.05. Scale bars: top panels = 3 mm; bottom panels = 400μm.

As previously reported [16], FGR was defined as a weight falling within the 10^th^ percentile (≤0.8071g on GD 17.5) of fetal weights calculated from a large pool obtained from saline-treated dams. In our earlier work, we showed that the LPS treatment regimen performed here lead to FGR with 40% of fetuses being growth restricted, and that pup weight increased significantly with GTN pre-treatment [16]. In the present study, placentas of growth-restricted fetuses exhibited significantly greater HIF-1α accumulation compared with placentas from non-growth restricted fetuses within the saline and LPS+GTN treatment groups (Fig 1A and 1B; p <0.05). Immunolocalisation of HIF-1α was mostly nuclear and not confined to any specific region of the placenta or maternal tissues. No significant differences in HIF-1α levels were observed between the LPS+GTN group and saline-treated controls (Fig 1A and 1B). In addition, we found that HIF-1α levels were negatively correlated with fetal weight (Fig 1C; r_s_ = -0.3989, p = 0.0290) across all treatment groups. To investigate a possible downstream consequence of augmented placental HIF-1α accumulation, we measured circulating levels of the vascular endothelial growth factor (VEGF) and placental growth factor antagonist, sFlt-1, in LPS-, saline-, and LPS+GTN treated rats but did not detect significant differences between these groups (S1 Fig).

### HIF-1α expression does not correlate with pimonidazole staining

**Fig 2:**
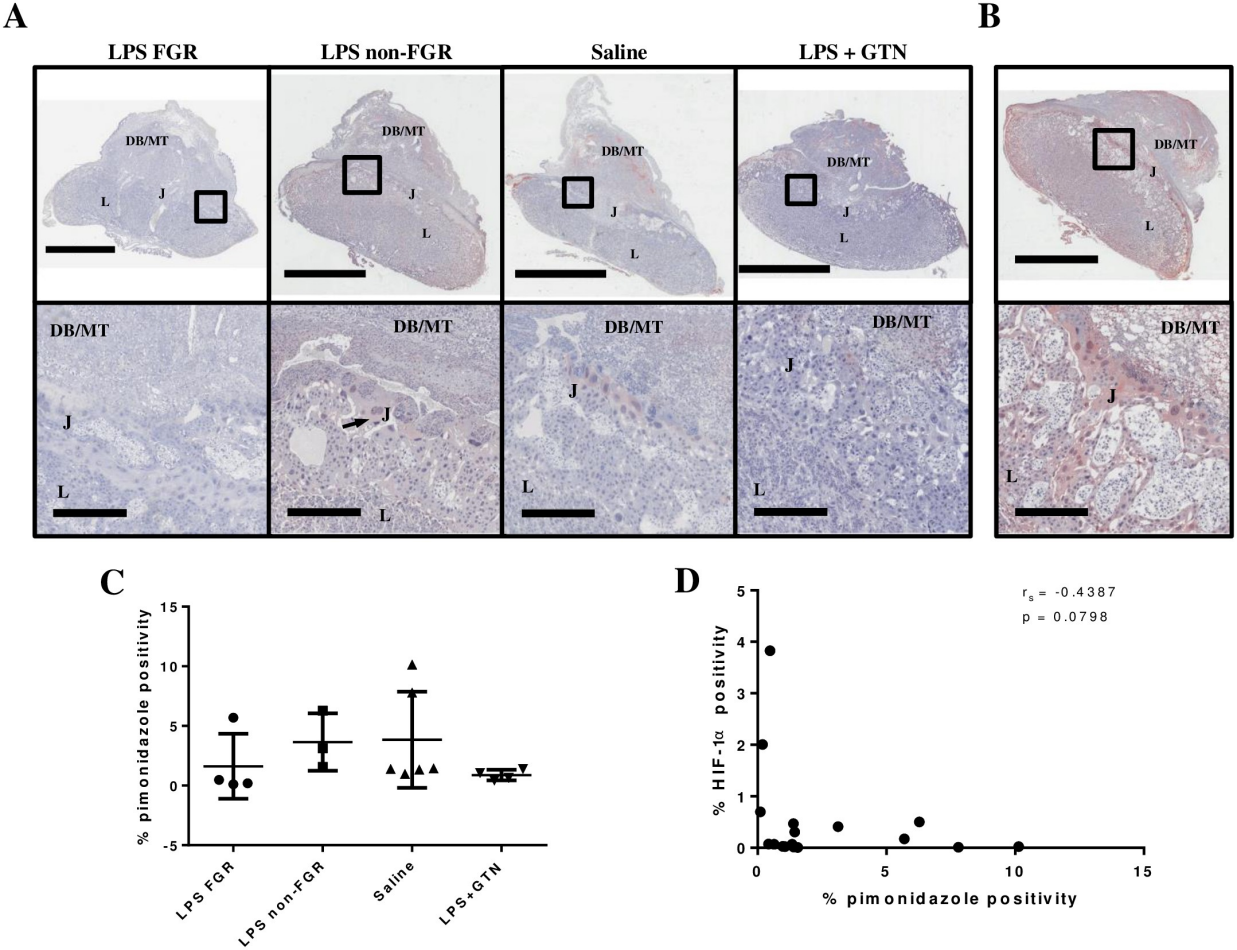
Placental HIF-1α expression does not correlate with pimonidazole staining. Placentas from all treatment groups exhibited similar levels of pimonidazole staining (red stain, arrows) (A) and no significant differences in the percentage area positively stained between groups (C). Sample images of a strongly stained section demonstrating intense immunostaining in trophoblast giant cells and the spongiotrophoblast layer of the junctional zone (B, arrows). There was no significant correlation between expression of HIF-1α and pimonidazole (n = 17; r_s_ = -0.4387; p = 0.0798; D). DB/MT, decidua basalis/mesometrial triangle; J, junctional zone; L, labyrinth. Scale bars: top panels (LPS/FGR, LPS/non-FGR, B), 3mm; top panels (Saline, LPS+GTN), 4mm; bottom panels, 400μm.

To assess whether HIF-1α accumulates in regions where there is detectable tissue hypoxia, we examined serial tissue sections from GD 17.5 implantation sites stained with antibodies against pimonidazole, to localize regions of hypoxia, and HIF-1α. In contrast to the increase in HIF-1α accumulation observed within the LPS-treated FGR group, no differences in levels of pimonidazole staining were seen between groups (Fig 2A and 2C). Most intense immunostaining of pimonidazole adducts was observed in the trophoblast giant cell layer and spongiotrophoblast layer of the junctional zone (Fig 2B) corroborating previous observations [30, 31]. We observed no correlation between the percentage of area stained for HIF-1α and pimonidazole. In fact, several placentas exhibiting clear HIF-1α immunostaining revealed low or undetectable pimonidazole staining (Fig 2D). We also noted that some areas showing HIF-1α expression did not stain positive for pimonidazole within serial sections.

## Discussion

**Fig 3:**
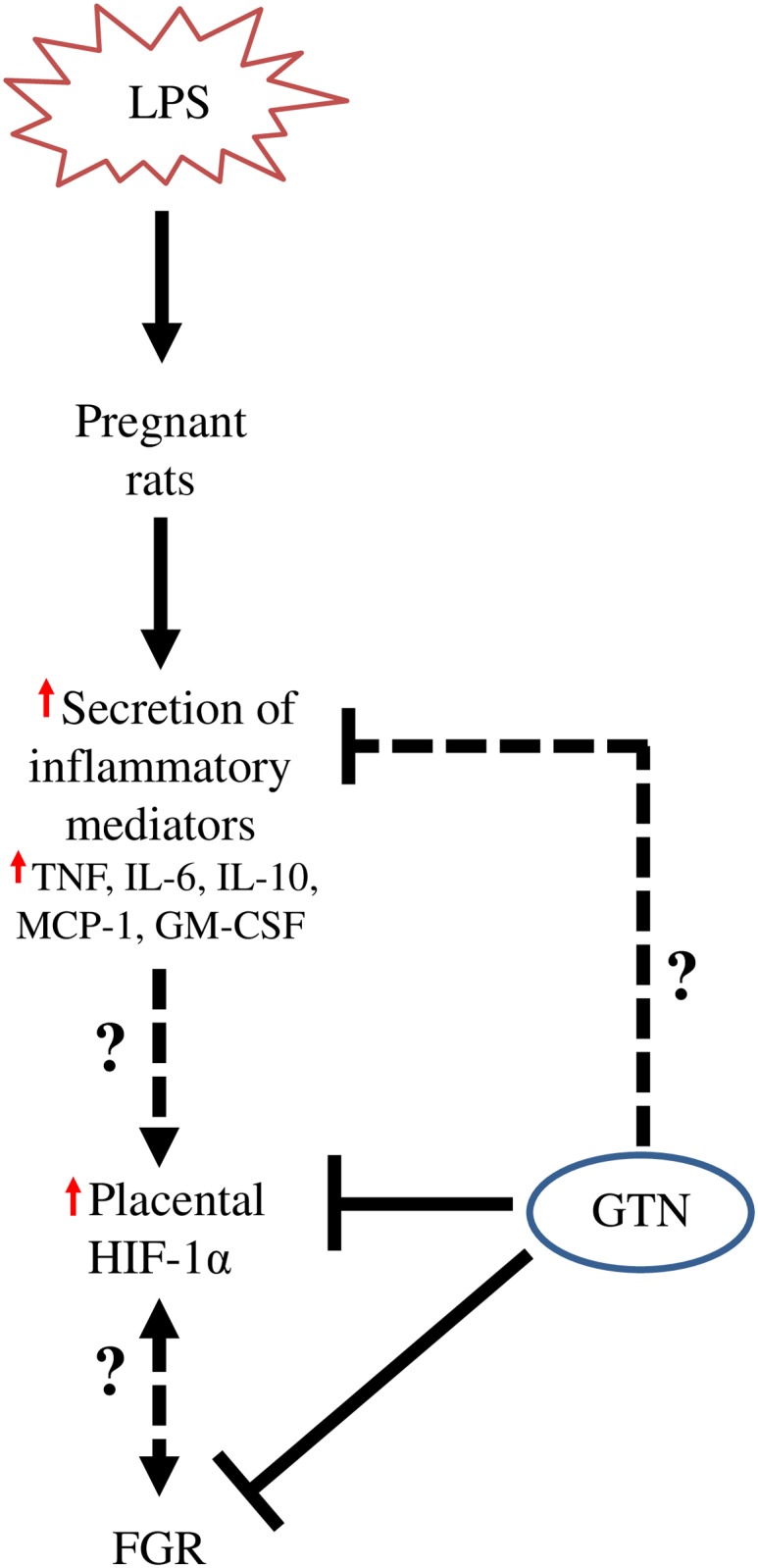
Proposed mechanism by which inflammation-induced FGR is linked to increased placental HIF-1α accumulation. Administration of LPS during pregnancy increases the secretion of a variety of inflammatory mediators including TNF, IL-6, IL-10, MCP-1, and GM-CSF. This heightened maternal inflammation contributes to increased HIF-1α accumulation, which in turn is associated with FGR. Administration of GTN prevents LPS-induced FGR and placental HIF-1α accumulation. A potential mechanism for this may involve suppression of inflammatory cytokine secretion by GTN. Further work is required to elucidate whether HIF-1α accumulation is secondary to FGR or vice versa.

The work presented here provides evidence that increased placental accumulation of HIF-1α is associated with the pathophysiology of inflammation-induced FGR. Our present study also reveals that the NO mimetic GTN prevented LPS-mediated increases in placental HIF-1α accumulation and confirms previous findings whereby GTN attenuated LPS-induced inflammation in pregnant rats [16]. A working model summarizing our findings is illustrated in Fig 3.

It is now well established that dysregulation of the maternal immune system can lead to pregnancy complications [32–34]. In human pregnancies affected by FGR, increased levels of inflammatory mediators have been reported in the maternal serum [35, 36] and cord blood [37, 38]. While normal pregnancy is considered to be a pro-inflammatory state, the degree of maternal inflammation may exist on a spectrum with heightened inflammation leading to pathological pregnancy [34, 39]. In fact, inflammatory stimuli that would normally be non-pathological in non-pregnant individuals are capable of inducing pregnancy complications [39, 40]. Our previously published data corroborate this observation since the effects of LPS were pregnancy-specific: in contrast to pregnant dams, LPS did not affect white blood cell counts in non-pregnant animals. Furthermore, non-pregnant rats did not exhibit the LPS-mediated renal structural alterations observed in pregnant rats, and TNF levels in non-pregnant rats treated with LPS were associated with only small increases in mean arterial pressure [16]. Moreover, we demonstrated a key role for TNF in mediating FGR, as inhibition of this inflammatory cytokine was shown to prevent FGR in LPS-treated dams [16]. Recognizing that a large number of inflammatory mediators contribute to FGR, the multiplex data shown here help to elucidate the effect of low-dose LPS administration on the maternal cytokine profile and provide further evidence for heightened maternal inflammation (Table 1). Many of the inflammatory factors that were found to be increased, including TNF, IL-6, IL-10, MCP-1, M-CSF and GM-CSF, have also been reported to be elevated in women experiencing pregnancy complications [34–36, 41, 42].

In the present study, HIF-1α accumulation was significantly increased in placentas of growth-restricted fetuses despite the absence of pimonidazole staining. Pimonidazole binding occurs only under conditions of severe hypoxia (pO_2_ levels less than 10 mmHg) [29], and a previous study demonstrated that the half-maximal expression of HIF-1α in HeLa cells subject to hypoxic conditions occurred between 1.5 and 2% O_2_ (11–15 mmHg) [43]. Therefore, it is possible that some of the HIF-1α accumulation observed was indeed due to low oxygen tension but at pO_2_ levels above the threshold for pimonidazole binding. However, there is also evidence that other factors can contribute to increased HIF-1α levels and these may also account for the placental HIF-1α accumulation reported here. The robust increase in pro-inflammatory molecules following LPS administration may be linked to increased HIF-1α accumulation mediated by NF-κB [19]. Moreover, inflammation-induced alterations in utero-placental perfusion resulting in activation of oxidative and nitrosative stress pathways [16] may be associated with HIF-1α expression [18, 44]. Importantly, oxidative stress and ROS production can occur in the absence of hypoxia since several factors, including inflammatory signalling pathways, can bring about this phenomenon [34, 45]. In addition, villous explants from pre-eclamptic placentas have been shown to display impaired oxygen-dependent degradation of HIF-1α [46, 47]; therefore, it is also possible that the HIF-1α over-expression observed in the FGR placentas is due to hypoxia occurring at an earlier time point accompanied by failure to adequately degrade HIF-1α. Thus, the observed accumulation of placental HIF-1α may be a result of one or, more likely, a combination of factors. Since our data reveal no differences in pimonidazole staining across all treatment groups despite an increase in placental HIF-1α accumulation within the LPS/FGR group, we believe that our finding showing a lack of correlation between HIF-1α expression and pimonidazole staining could indicate that HIF-1α may be upregulated by mechanisms other than hypoxia during this late stage of pregnancy.

Interestingly, our data revealed that GTN, a known vasodilator and NO mimetic, prevented LPS-induced increases in placental HIF-1α, supporting our previous study whereby GTN attenuated hypoxia-mediated accumulation of HIF-1α in human term chorionic villi explants [24]. Reduced NO bioavailability is associated with FGR in humans [48–50], and in our earlier work, we showed that GTN prevented inflammation-induced FGR by reducing spiral artery resistance index, placental nitrosative stress, mean arterial pressure and renal structural alterations using the same rat model described here [16]. In another model of FGR, endothelial nitric oxide synthase (eNOS) deficiency has been shown to cause hemodynamic and vascular changes that were linked to placental hypoxia [31, 51, 52]. However, in our inflammation-induced FGR model, the impaired spiral artery remodelling observed [16] does not necessarily indicate that placental hypoxia would occur since remodelling and subsequent dilation of the spiral arteries alone is thought to have a minimal effect on total blood flow [53]. There is recent evidence that failed spiral artery remodelling may not cause local hypoxia as a result of decreased blood volume delivered to the fetal-maternal interface; rather, high-momentum blood entering the intervillous space and/or ischemia/reperfusion injury leading to local tissue damage may be the source of placental pathology in FGR/PE [53]. Since the uterine and radial arteries lie upstream and are rate-limiting vessels, rheological changes in the spiral arteries rather than hypoxia could lead to placental damage [53]. Given our findings suggesting that the placenta is adequately oxygenated, flow through the uterine and radial arteries may be unaffected by our LPS treatment regimen. This would allow total blood flow to be maintained while the inadequately remodelled spiral arteries observed in our model [16] may shoot concentrated jets of blood that damage the placental villi. The therapeutic effect of GTN could therefore be attributed to the vasodilation of these spiral arteries, which would aid in normalising blood flow.

Alternatively, it is possible that GTN prevents HIF-1α accumulation and FGR by targeting inflammatory signalling pathways directly. *In vitro* studies conducted in our laboratory have shown that pre-treatment of THP-1 macrophages with sub-micromolar concentrations of GTN prevents TNF release in conditioned media collected four hours after LPS stimulation, suggesting that GTN may possess anti-inflammatory functions independent of its vasodilatory effects (unpublished work). Moreover, in our model of inflammation-induced PE/FGR, GTN treatment normalised white blood cell counts in LPS-treated pregnant rats [16]. While we were not able to observe any differences in the cytokine profile of the LPS+GTN group compared to either the saline- or LPS-treated dams, this is perhaps because we examined only a two-hour timepoint post-LPS administration and were therefore unable to assess temporal regulation of these cytokines.

This study provides evidence for a novel link between HIF-1α and the pathophysiology of FGR; however, the consequences of placental HIF-1α accumulation require further study. HIF-1α has been shown to be capable of inducing increases in circulating sFlt-1 and sEng, two key factors involved in the pathogenesis of FGR [54, 55]. Indeed, our group showed previously that low concentrations of GTN greatly attenuated hypoxia-induced sFlt-1 release from human term chorionic villi explants through a potential mechanism involving HIF-1α inhibition [24]. While we were not able to detect differences in circulating sFlt-1 levels between the LPS, saline, or LPS+GTN treated rats, this is perhaps because not all the placentas and implantation sites in LPS-treated rats showed evidence of placental HIF-1α accumulation or FGR, thus diluting the effect of increased HIF-1α in individual implantation sites on systemic levels of sFlt-1. Here, we propose that increased placental HIF-1α accumulation is linked to inflammation-induced FGR and may be associated with the pathogenesis of this condition. Our finding that GTN prevents inflammation-induced HIF-1α accumulation associated with FGR requires further study but may represent a novel therapeutic opportunity.

## Supporting information

S1 Fig Circulating sFlt-1 levels in the three treatment groups. No significant differences in sFlt-1 levels were observed between the LPS (N = 10 dams), Saline (N = 8), or LPS+GTN (N = 7) groups at GD 17.5.
(PPTX)

S1 File Supporting data values for Fig 1. (XLSX)

S2 File Supporting data values for Fig 2. (XLSX)

S3 File Supporting data values for S1 Fig. (XLSX)
