# Supplementary figures and images for: Inflammation-induced fetal growth restriction in rats is associated with increased placental HIF-1α accumulation
Source: PLoS One. 2017 Apr 19;12(4):e0175805. doi: 10.1371/journal.pone.0175805 (PMC5397034; doi:10.1371/journal.pone.0175805)

## Slide 1
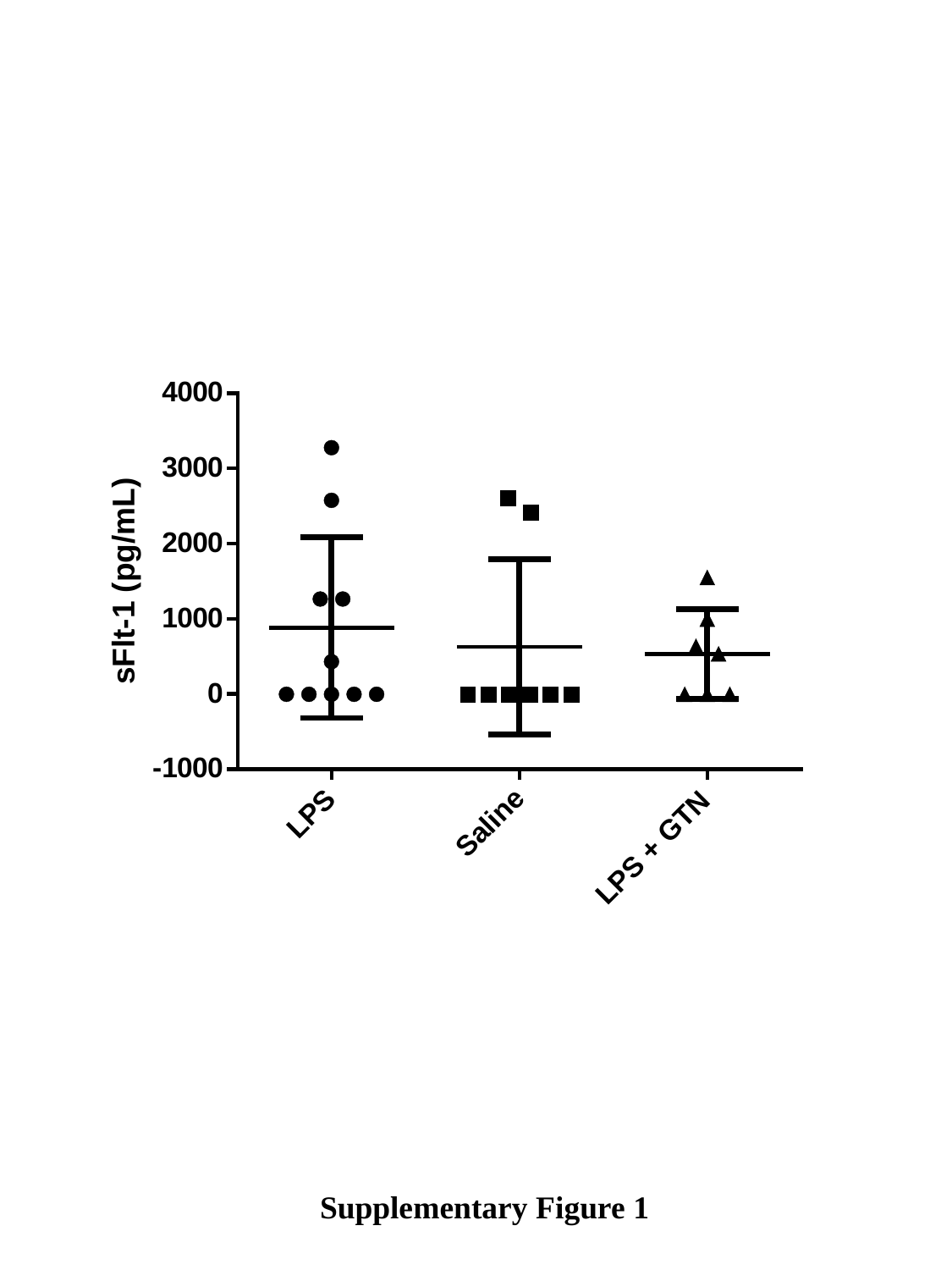

Supplementary Figure 1

Supplement: S1 Fig — No significant differences in sFlt-1 levels were observed between the LPS (N = 10 dams), Saline (N = 8), or LPS+GTN (N = 7) groups at GD 17.5. (PPTX) [file pone.0175805.s001.pptx]
